# Bacterial clusters are associated with the risk of severe disease progression in inflammatory bowel disease irrespective of conventional disease categories

**DOI:** 10.20517/mrr.2025.96

**Published:** 2026-03-18

**Authors:** Simen Hyll Hansen, Niharika Bhattacharjee, Chang Hu, Maria Gjerstad Maseng, Olle Grannö, Corinna Bang, Christine Olbjørn, Gøri Perminow, Jørgen Valeur, May-Bente Bengtson, Svein Oskar Frigstad, Svend Andersen, Tone Bergene Aabrekk, Trond Espen Detlie, Andre Franke, Vendel A. Kristensen, Jonas Halfvarson, Marte Lie Høivik, Ravishankar K. Iyer, Johannes Hov

**Affiliations:** ^1^Norwegian PSC Research Center, Department of Transplantation Medicine, Division of Surgery, Inflammatory Diseases and Transplantation, Oslo University Hospital, Oslo 0372, Norway.; ^2^Institute of Clinical Medicine, Faculty of Medicine, University of Oslo, Oslo 0372, Norway.; ^3^Research Institute of Internal Medicine, Division of Surgery, Inflammatory Diseases and Transplantation, Oslo University Hospital, Oslo 0372, Norway.; ^4^Siebel School of Computing and Data Science, University of Illinois Urbana-Champaign, Urbana-Champaign, IL 61820, USA.; ^5^Department of Electrical and Computer Engineering, University of Illinois Urbana-Champaign, Urbana-Champaign, IL 61820, USA.; ^6^Department of Gastroenterology, Oslo University Hospital, Oslo 0450, Norway.; ^7^Bio-Me, Oslo 0349, Norway.; ^8^Department of Laboratory Medicine, Clinical Microbiology, Faculty of Medicine and Health, Örebro University, Örebro 70182, Sweden.; ^9^Institute of Clinical Molecular Biology, Christian-Albrechts-University of Kiel, Kiel 24118, Germany.; ^10^Department of Paediatric and Adolescent Medicine, Akershus University Hospital, Lørenskog 1478, Norway.; ^11^Department of Pediatrics, Oslo University Hospital, Oslo 0372, Norway.; ^12^Unger-Vetlesen Institute, Lovisenberg Diaconal Hospital, Oslo 0456, Norway.; ^13^Department of Gastroenterology, Vestfold Hospital Trust, Tønsberg 3103, Norway.; ^14^Department of Medicine, Bærum Hospital, Vestre Viken Hospital Trust, Gjettum 1346, Norway.; ^15^Department of Paediatrics, Vestfold Hospital Trust, Tønsberg 3103, Norway.; ^16^Medical Department, Vestfold Hospital Trust, Tønsberg 3103, Norway.; ^17^Department of Gastroenterology, Akershus University Hospital, Lørenskog 1478, Norway.; ^18^Department of Gastroenterology, Faculty of Medicine and Health, Örebro University, Örebro 70182, Sweden.; ^19^Section of Gastroenterology, Department of Transplantation Medicine, Division of Surgery, Inflammatory Diseases and Transplantation, Oslo University Hospital, Oslo 0372, Norway.; ^#^These authors contributed equally to this work and shared first authorship.; ^†^These authors contributed equally to this work and shared senior authorship.

**Keywords:** Microbiome, clustering, IBD, prognosis, GMM

## Abstract

**Background:** Inflammatory bowel diseases (IBDs) are complex conditions marked by chronic inflammation in the gastrointestinal tract. Traditional classification separates IBD into Crohn’s disease and ulcerative colitis, but this division may not fully capture disease heterogeneity. Here, we examine whether microbiome-driven subtyping can describe novel clinical IBD phenotypes. To achieve this, we applied unsupervised clustering to fecal microbiota profiles from the population-based Inflammatory Bowel Disease in South-Eastern Norway III (IBSEN III) cohort.

**Methods:** A Gaussian Mixture Model (GMM) was used to cluster participants with IBD based on microbiome composition and examine associations between clusters and clinical outcomes, including inflammatory markers and disease severity during the first year after inclusion.

**Results:** Three microbiome-based clusters were identified: CLO (dominated by *Clostridia UCG-014*), ALF (*Agathobacter, Lachnoclostridium*, and *Faecalibacterium*), and RUM (*Ruminococcus gnavus*). Participants in the RUM cluster had a higher risk of future severe disease than those in the CLO cluster, even among participants with remission-to-mild disease at inclusion (21% *vs.* 6%, *P* < 0.00001). This association could not be explained by antibiotic use or baseline disease severity. Cluster membership alone performed comparably to fecal calprotectin in distinguishing severe disease, and a combined model significantly improved accuracy (*P* < 0.0001).

**Conclusion:** Our findings demonstrate a connection between microbiome composition and the risk of severe disease development, which is partly independent of inflammation levels at the time of sampling. Microbiome-informed subgrouping could lead to more personalized treatment strategies. Further validation is needed to determine the clinical utility of these clusters.

## INTRODUCTION

Inflammatory bowel disease (IBD) includes two main diagnostic subtypes, ulcerative colitis (UC) and Crohn’s disease (CD), both characterized by chronic inflammation of the gastrointestinal tract. A leading hypothesis is that IBD is caused or maintained by an inappropriate immune response to gut bacteria^[1,2]^. This immune response may alter the microbial composition both by selectively targeting specific microbes and by reducing the survival of bacterial lineages that are less capable of persisting in chronically inflamed, oxygen-enriched environments^[3,4]^. Both the relative abundance of specific bacterial species and the overall composition of the IBD microbiome differ from those of healthy people. However, no compelling evidence currently exists for the exact mechanisms behind these inappropriate immune responses to the gut microbiome. 

Currently, diagnosis and subclassification of IBD are still based on symptoms, endoscopic, histological and radiological findings. However, these conventional categories only partially capture the highly heterogeneous clinical presentations and disease courses of these lifelong conditions and provide limited guidance for medical treatment decisions^[5]^. If the microbiome is tied to IBD pathogenesis, its composition may affect the efficacy of different treatments. Improved molecular and microbial subclassification could therefore facilitate improved diagnostics, treatment stratification, and targeted therapeutics. 

Genetic gradients are well known to partly underlie variation in disease distribution in IBD^[6]^, suggesting that current classification systems may represent arbitrary cutoffs along a continuum. The altered IBD microbiome varies according to specific subtypes (i.e., CD and UC)^[7,8]^, and our recent study indicates that the gut microbiome also varies with disease distribution^[9]^. Additionally, studies have shown associations between microbial composition and the future risk of severe disease^[8,10]^. Together, these findings indicate that the fecal microbiome has potential diagnostic and prognostic value and may be involved in the phenotypic determination of IBD.

Given the clinical and molecular heterogeneity of IBD and the clinical need for improved classification, we explored whether sequencing-based microbiome profiles could reclassify IBD into new subgroups. Our aim was to provide stable cluster assignments with high confidence and to describe these patient clusters with regard to disease features and outcomes, thereby demonstrating their clinical relevance. Specifically, we hypothesized that microbiome profiles could be used to describe clinically meaningful subgroups within IBD, independent of traditional disease categories.

## METHODS

### Participants and data collection

We used data from the well-defined IBSEN III cohort (Inflammatory Bowel Disease in South-Eastern Norway)^[11]^. This population-based inception cohort recruited participants of all ages referred with suspected IBD between 2017 and 2019 in the South-Eastern Norway health region. The study design, inclusion criteria, and diagnostic procedures are described in detail by Kristensen *et al.*^[11]^. In the current study, we included 970 patients from the IBSEN III cohort, all of whom were diagnosed with IBD after a diagnostic work-up including endoscopy. At the time of inclusion (baseline), clinical disease activity was recorded using the simple clinical colitis activity index (SCCAI) for UC^[12]^ and the Harvey-Bradshaw index (HBI) for CD^[13]^. Patients with incomplete clinical metadata were excluded from the analyses. Subtype-specific Montreal classification was included in some analyses. Antibiotic use within 3 months prior to study inclusion was recorded, and samples from participants with such antibiotic exposure were excluded from specific sensitivity analyses.

At inclusion, participants received two collection tubes. Fecal samples for microbiota analysis were collected in tubes containing DNA stabilizer (Invitek Diagnostics, Berlin, Germany), while a dry tube was used for fecal calprotectin (F-calprotectin) measurement. Samples were shipped by participants at ambient temperature and, upon receipt, were stored at -80 °C. Samples were subsequently prepared for polymerase chain reaction (PCR) amplification of the V3-V4 region of the 16S rRNA gene according to a standard protocol^[14]^. After pooling of amplicons, sequencing was performed using the Illumina MiSeq platform and v3 kit (Illumina) at the Norwegian Sequencing Centre, Oslo. Samples yielding fewer than 10,000 reads were resequenced at least once. Samples that did not reach > 10,000 reads after at least two rounds of sequencing were excluded from the current study. F-calprotectin was analyzed using the Bühlmann Calprotectin ELISA EK-CAL kit (Schönenbuch, Switzerland), with results recorded within the range of 30-1800 μg/g. Values outside this range were set to +/- 1 of the range’s lower or upper limit.

### Ethics

This study is a secondary analysis of data from the IBSEN III cohort. The IBSEN III study was approved by the Regional Committee for Medical and Health Research Ethics in South-Eastern Norway (REK) (IRB number: 2015/946). The study was conducted in accordance with the Declaration of Helsinki, and data handling complied with applicable data protection regulations, including approval through a Data Protection Impact Assessment (DPIA). Written informed consent was obtained from all participants at the time of enrollment in the IBSEN III cohort.

### Bioinformatics

The full bioinformatics pipeline is described in detail in the Supplementary Methods section. Briefly, the sequences were filtered, demultiplexed, trimmed, and merged using *bbduk*,* cutadapt*, and *bbmerge*^[15-17]^. Next, they were denoised using Deblur as implemented in *QIIME2 2021.4* and classified against the SILVA 138 database^[18-21]^. Contaminants were removed before the remaining sequences were collapsed to the genus level and exported from *QIIME2 *for subsequent analysis and modeling. Clusters were computed with *Python *3.8.5^[22]^ using the *scikit-learn 1.3.0*^[23]^ package, and initial visualizations were generated using *pca 2.0.5*, *seaborn 0.11.0*, and *pandas 2.0.3*. Analysis of clusters was done in R *4.2.3*^[24]^ and visualized with the R package *ggplot2 3.4.1*^[25]^.

### Statistics

The Shapiro-Wilk test was used to check for normality of continuous variables, followed by Tukey’s test for pairwise tests of normal data and Dunn’s test for non-normal data. Dunn’s test was also applied for pairwise testing following a Kruskal-Wallis test for ordinal numerical variables, such as the Bristol Stool Scale. The chi-square test was used in conjunction with proportion analysis for binary and categorical variables. Dominant bacteria per cluster were defined as the number of appearances in filtered lists of taxa with stepwise increasing abundance (0 to 1, in steps of 0.001) and prevalence (0.5 to 1, in steps of 0.01).

### Baseline severity

To minimize the introduction of bias in the analysis pipeline with respect to the traditional UC-CD dichotomy, diagnostically specific scores were merged. Specifically, baseline clinical disease severity was assessed using SCCAI for UC and HBI for CD. The two scoring systems were converted to a common four-level scale, based on common classifications of the scores into symptomatic remission, mild, moderate, and severe disease^[26,27]^. In short, remission was defined as SCCAI < 3 or HBI < 5, mild disease as SCCAI 3-5 or HBI 5-7, moderate disease as SCCAI 6-11 or HBI 8-16, and severe disease as SCCAI > 11 or HBI > 16. Pediatric participants were not included in this common scale and were excluded from analyses requiring baseline severity as input.

### Severe disease course

We used an already published objective definition of a severe disease course^[28]^. Participants were classified as having a severe disease course if they met one or more of the specific criteria within the first year after study inclusion. For UC, these criteria included IBD-related hospitalization, IBD-related surgery, use of more than two courses of corticosteroids, or more than one course of biologic medications. For CD, the same criteria applied, plus additional criteria including the development of fistulas, abscesses, or strictures, or surgery for these complications.

### Clustering methodology

The sequencing dataset was compositional in nature. Each patient was represented by 149 microbial features, with values reflecting the relative abundances of different microbial taxa. To reduce noise and prioritize biologically relevant features, those not present in at least 12% of samples were filtered out and the remaining 137 features were renormalized to sum to 1. The 12% cutoff was chosen as the most conservative threshold that retained the maximal number of features while still producing well-separated points in the ordination space (prior to clustering). After feature selection, the compositional data analysis method known as the centered log-ratio (CLR) transformation was applied, in which the transformed data for each patient was expressed as log ratios of each feature relative to the geometric mean of all features^[29]^. The CLR transformation was essential for projecting microbiome data, which are compositional, zero-inflated, and non-normal, into real-valued Euclidean space. This allowed the application of standard statistical methods, including Principal Component Analysis (PCA) and varied types of clustering methods^[30]^. Several clustering methods were evaluated on the CLR-transformed dataset with the objective of identifying stable clusters that can provide clinically meaningful insights. The methods tested are described in detail in the Supplementary Methods section, and include Partitioning Around Medoids (PAM), Agglomerative Clustering (Ward’s Method), Spectral Clustering, and Gaussian Mixture Model (GMM) clustering. Additionally, Dirichlet Multinomial Mixture (DMM) clustering was benchmarked for k = 3 using raw counts instead of CLR-transformed data. PCA was used to verify that each clustering method produced output consistent with the overall structure and applied normalization techniques. The stability of each clustering method was assessed by calculating the average Adjusted Rand Index (ARI) across multiple bootstrap replicates. For each clustering method, the cluster stability score represented the average ARI over all pairs of bootstrap samples. A cluster stability score of 1 indicated perfect agreement between cluster assignments across the bootstrap samples, whereas a score of 0 indicated that the assignments were no better than random. To evaluate cluster compactness and separation, the Silhouette score was computed across clustering methods. Specifically, for GMM, the Akaike information criterion (AIC) and Bayesian information criterion (BIC) were used to determine the optimal number of components (clusters) in the range k = 2-9. 

## RESULTS

In total, 1,121 individuals with baseline fecal microbiota profiles were eligible for inclusion in this study. Of these, 151 were excluded due to incomplete clinical metadata [Supplementary Table 1], resulting in a final study population of 970 participants with IBD. The average age was 37; 10% were < 18 years old at inclusion, and 52% of participants were female. Among the evaluated clustering methods suitable for zero-inflated, non-normally distributed data subject to transformation, clusters based on GMM demonstrated the highest stability (*ARI = 0.74, *Supplementary Table 2). Considering the number of clusters, k = 3 was identified and selected as the optimal number of components for GMM clustering [Supplementary Figure 1].

### Demographic and microbial characterization of new IBD clusters

All participants were assigned to one of three clusters: cluster 1 (*n* = 408), cluster 2 (*n* = 355), and cluster 3 (*n* = 207). Participants in cluster 1 were older on average, while cluster 2 had a larger proportion of males [Table 1]. 

**Table 1:** Summary of key demographics (age, sex, body mass index) and confounders (antibiotic use and Bristol Stool Scale)

	**CLO** **(*n* = 408)** **[Cluster 1]**	**ALF** **(*n* = 355)** **[Cluster 2]**	**RUM** **(*n* = 207)** **[Cluster 3]**	**Total** **(*n* = 970)**
Age (years) [Mean (95% CI)]	41 (40-43)	33 (32-35)	35 (33-37)	37 (36-38)
Pediatric (<18 years)	27 (7%)	43 (14%)	28 (16%)	98 (10%)
Sex (female)	226 (55%)	156 (44%)	121 (59%)	503 (52%)
Body Mass Index [Mean (95% CI)]	25 (24-25)	25 (24-25)	25 (24-26)	25 (24-25)
Missing	5 (1%)	7 (2%)	7 (3%)	19 (2%)
Antibiotics*	16 (4%)	27 (8%)	48 (23%)	91 (9%)
Bristol Stool Scale [Mean (95% CI)]	4 (3.7-4.1)	4 (3.9-4.4)	5 (4.3-5.0)	4 (4.0-4.3)
Missing	193 (47%)	188 (53%)	102 (49%)	483 (50%)

Detailed tables with statistical results are included in the Supplementary Materials [Supplementary Table 3]. *Antibiotic usage within 3 months prior to study inclusion. CI: Confidence interval; CLO:* Clostridia UCG-014*; ALF: *Agathobacter, Lachnoclostridium*, and *Faecalibacterium*; RUM: *Ruminococcus gnavus*.

**Figure 1:**
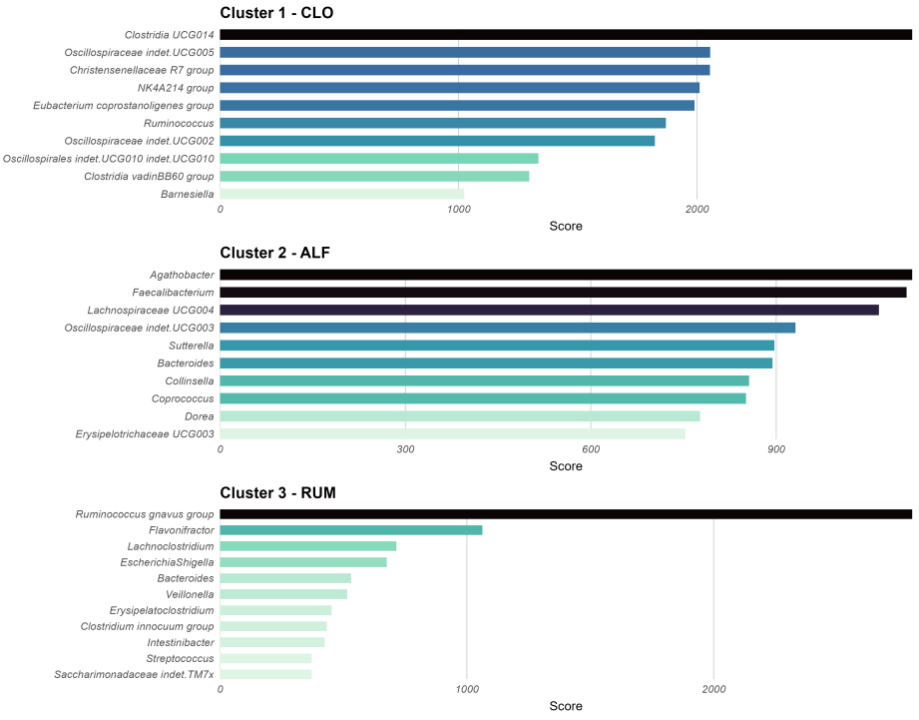
Summary of top-ranking taxa, calculated as the number of presences across a range of abundance and prevalence thresholds (see Methods). This plot was used to name the clusters in this study. The order of clusters in the plot is: 2, 1, 3. Cluster 2 exhibited co-dominance of several taxa (Agathobacter, Lachnospiraceae UCG-004, and Faecalibacterium), leading to the name ALF based on the initials. Cluster 1 was dominated by Clostridia UCG-014, giving the name CLO, and Cluster 3 was dominated by Ruminococcus gnavus, leading to the name RUM. Figure created with ggplot2.0 in R. CLO:* Clostridia UCG-014*; ALF: *Agathobacter, Lachnoclostridium*, and *Faecalibacterium*; RUM: *Ruminococcus gnavus*.

**Figure 2:**
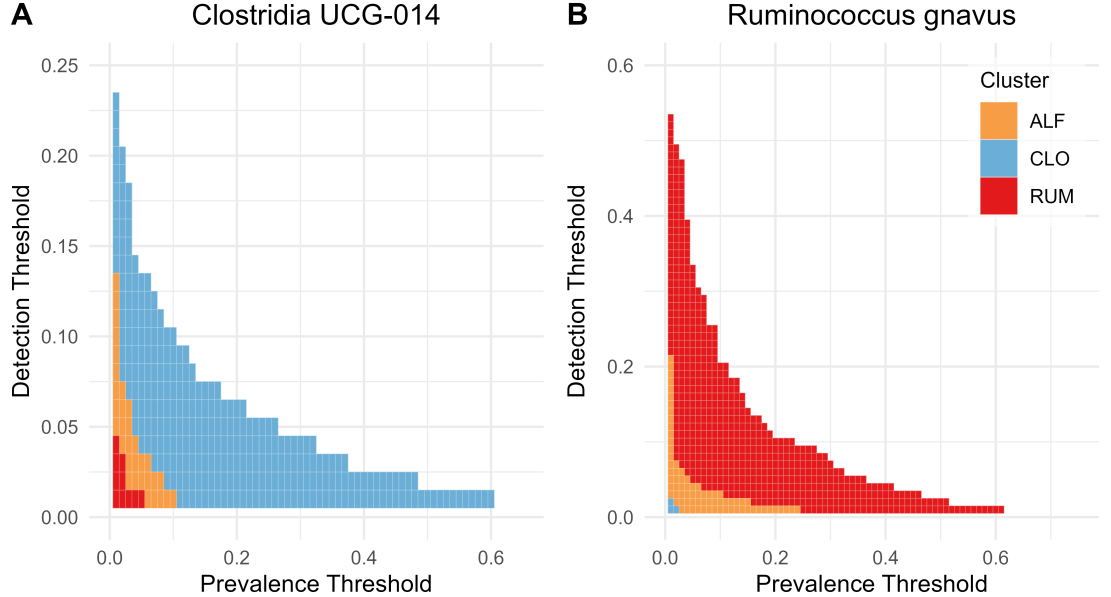
Heatmaps highlighting differences in prevalence and abundance (detection threshold) between clusters for (A) Ruminococcus gnavus and (B) Clostridia UCG-014. Blue = CLO, yellow = ALF, red = RUM. Figure created with ggplot2.0 in R. CLO:* Clostridia UCG-014*; ALF: *Agathobacter, Lachnoclostridium*, and *Faecalibacterium*; RUM: *Ruminococcus gnavus*.

Each cluster exhibited a unique set of the most prevalent and abundant bacterial genera [Figure 1]. The clusters were named according to their top genera: cluster 1 was designated as “CLO” due to the high prevalence of *Clo**stridia UCG-014, *cluster 3 as “RUM” due to the high abundance of *Ruminococcus gnavus*, while cluster 2 was named “ALF” because its top-ranking taxa were *A**gathobacter, **L**achnoclostridium UCG-004*, and *Faecalibacterium*. While these defining taxa were not exclusive to their respective clusters, their prevalence and abundance were clearly higher in these clusters, as shown for RUM and CLO in Figure 2A and B. 

**Figure 3:**
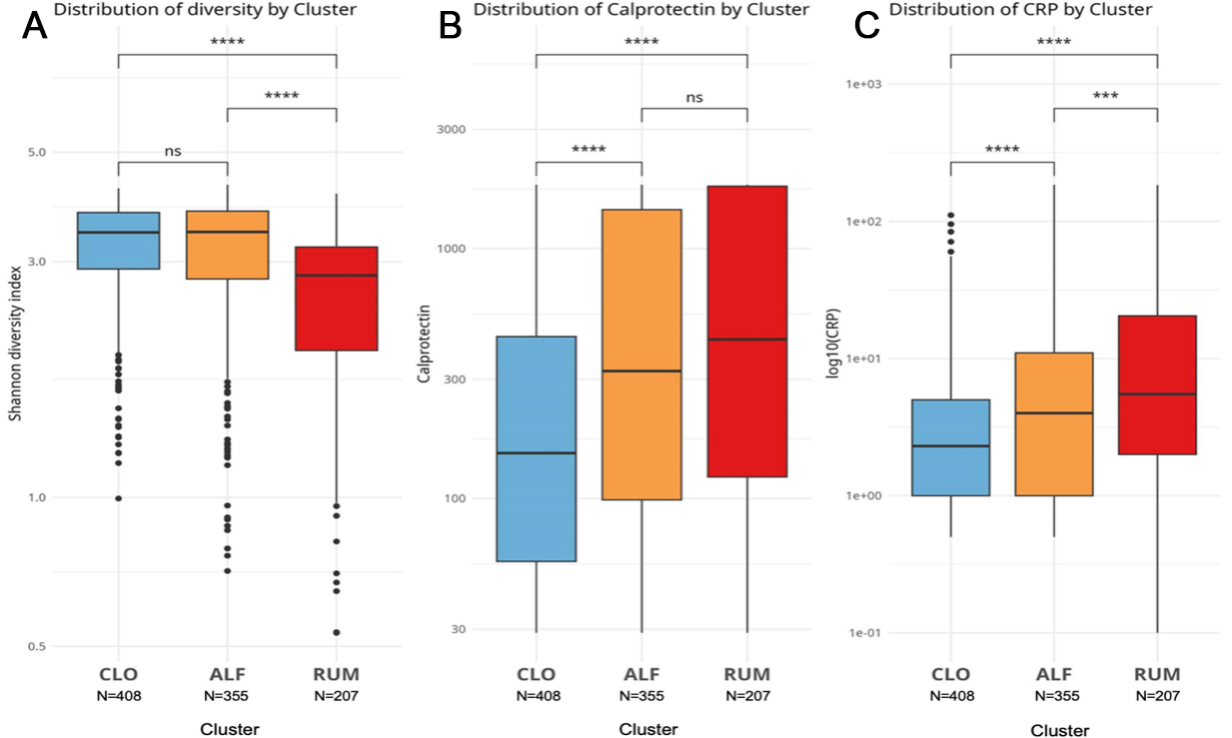
Boxplots comparing key measurements between clusters, with Mann-Whitney test statistics: (A) Shannon diversity index, (B) fecal calprotectin, and (C) C-reactive protein. Blue = CLO, yellow = ALF, red = RUM. Ns = not significant, ***P < 0.001, ****P < 0.0001. Figure created with ggplot2.0 in R. CLO:* Clostridia UCG-014*; ALF: *Agathobacter, Lachnoclostridium*, and *Faecalibacterium*; RUM: *Ruminococcus gnavus*; CRP: C-reactive protein.

**Figure 4:**
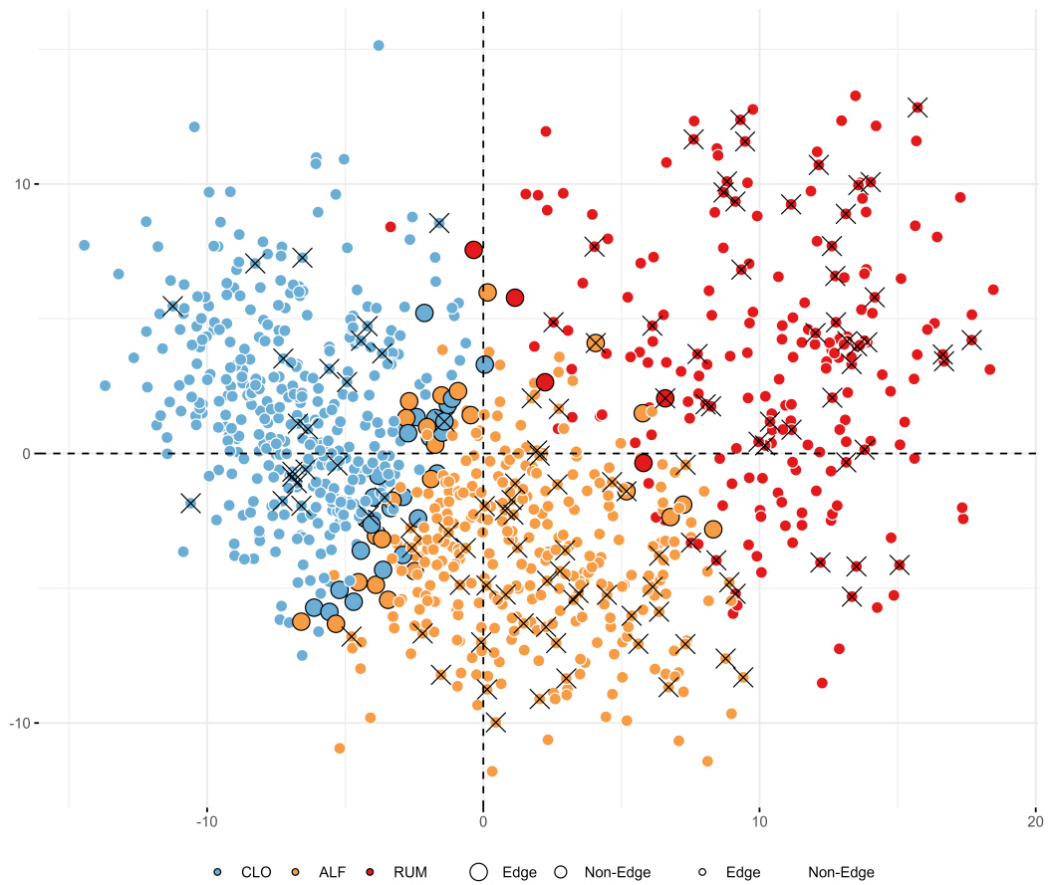
Beta diversity plot based on Bray-Curtis distances of relative abundances, highlighting cluster assignments (blue = CLO, yellow = ALF, red = RUM), cases of severe disease course during the first year (crosses), and samples that could not be confidently (> 90%) assigned to any cluster (larger dots with black outlines). Variance explained by PC1 = 13%, PC2 = 6%. Figure created with ggplot2.0 in R. CLO:* Clostridia UCG-014*; ALF: *Agathobacter, Lachnoclostridium*, and *Faecalibacterium*; RUM: *Ruminococcus gnavus*; PC: principal coordinates.

With regard to intraindividual (alpha) microbial diversity, the Shannon diversity index was lower in RUM compared with the other clusters (Figure 3A, *P < 0.0001*). Considering cluster assignments as categorical variables in a global microbiota analysis (beta diversity) based on the Bray-Curtis dissimilarity index, which accounts for overlap in relative abundances of taxa, the clusters occupied distinct regions of the complete sample set, with minimal overlap (Figure 4, *P < 0.001* for all pairwise ADONIS tests).

RUM had the highest rate of antibiotic usage within the 3 months preceding study inclusion (23% *vs.* 4% and 8% in CLO and ALF, respectively; *P < 0.0001*, chi-square test). A sensitivity analysis excluding participants with antibiotic exposure yielded highly similar clusters, both in terms of top-ranking taxa [Supplementary Figure 2] and general demographics, rates of severe disease course, and clinical characteristics [Supplementary Table 3].

### Clinical features of the IBD clusters

ALF and RUM had higher levels of F-calprotectin and C-reactive protein (CRP) compared with CLO [Figure 3B and C], with RUM tending to exhibit the highest levels. Baseline disease severity scores (based on categorizations of SCCAI and HBI; see METHODS) were also higher in RUM compared to ALF and CLO (Dunn’s test, *P *= 0.011 and *P *< 0.0001*, *respectively; Table 2). Regarding the location of inflammation, the clusters followed a pattern of increasing extension and severity (based on Montreal classifications in adults) in the order: mild in CLO, medium in ALF, and severe in RUM [Table 2]. The most common disease phenotype in CLO was ulcerative proctitis, while extensive (total) colitis was predominant in ALF and RUM. The rate of ileal inflammation, however, was comparable in all clusters (chi-square test, *P* > 0.05 for all comparisons; Table 2). 

**Table 2:** Cluster-wise overview of key clinical characteristics

	**CLO** **(*n* = 408)**	**ALF** **(*n* = 355)**	**RUM** **(*n* = 207)**	**Overall** **(*n* = 970)**
**IBD diagnosis**				
UC	275 (67%)	234 (66%)	116 (56%)	625 (64%)
CD	133 (33%)	121 (34%)	91 (44%)	345 (36%)
**Disease course**				
Indolent	385 (94%)	306 (86%)	161 (78%)	852 (88%)
Severe	23 (6%)	49 (14%)	46 (22%)	118 (12%)
**UC Montreal Extension**				
Ulcerative proctitis	129 (47%)	78 (33%)	20 (17%)	227 (36%)
Left-sided	83 (30%)	40 (17%)	21 (18%)	144 (23%)
Extensive	53 (19%)	98 (42%)	59 (51%)	210 (34%)
Missing	10 (4%)	18 (8%)	16 (14%)	44 (7%)
**UC Montreal Severity**				
Clinical remission	15 (5%)	5 (2%)	1 (1%)	21 (3%)
Mild	119 (43%)	71 (30%)	21 (18%)	211 (34%)
Moderate	115 (42%)	123 (53%)	58 (50%)	296 (47%)
Severe	16 (6%)	16 (7%)	20 (17%)	52 (8%)
Missing	10 (4%)	19 (8%)	16 (14%)	45 (7%)
**CD Montreal Location**				
Ileal	60 (45%)	37 (31%)	36 (40%)	133 (39%)
Colonic	22 (17%)	20 (17%)	16 (18%)	58 (17%)
Ileocolonic	27 (20%)	34 (28%)	20 (22%)	81 (23%)
Upper disease modifier	2 (2%)	4 (3%)	1 (1%)	7 (2%)
Missing	18 (14%)	26 (21%)	16 (18%)	60 (17%)
**CD Montreal Behavior**				
Non-stricturing, non-penetrating	93 (70%)	76 (63%)	53 (58%)	222 (64%)
Stricturing	15 (11%)	14 (12%)	14 (15%)	43 (12%)
Penetrating	0	1 (1%)	2 (2%)	3 (1%)
Perianal disease modifier	1 (1%)	3 (2%)	4 (4%)	8 (2%)
Missing	18 (14%)	26 (21%)	16 (18%)	60 (17%)
**UC SCCAI** [Mean (95% CI)]	2.6 (2.3-3.0)	3.2 (2.8-3.6)	4.3 (3.7-4.9)	3.2 (2.9-3.4)
**CD HBI** [Mean (95% CI)]	3.2 (2.8-3.6)	4.7 (3.9-5.4)	4.7 (4.0-5.3)	4.1 (3.7-4.5)
**F-calprotectin ** [Mean (95% CI)]	393 (342-444)	675 (602-748)	770 (673-868)	577 (535-618)
Missing	1 (0.2%)	1 (0.3%)	1 (0.5%)	3 (0.3%)
**C-reactive protein** [Mean (95% CI)]	6 (5-7)	11 (9-14)	19 (14-23)	11 (9-12)
Missing	19 (4.7%)	25 (7.0%)	8 (3.9%)	52 (5.4%)

Detailed statistical results are provided in the Supplementary Materials [Supplementary Table 3]. Missing data largely correspond to pediatric participants, who were assessed using different classification and scoring systems and are therefore not included in this table. IBD: Inflammatory bowel disease; CD: Crohn’s disease; UC: ulcerative colitis; SCCAI: simple clinical colitis activity index; HBI: Harvey-Bradshaw index; CI: confidence interval; CLO:* Clostridia UCG-014*; ALF: *Agathobacter, Lachnoclostridium*, and *Faecalibacterium*; RUM: *Ruminococcus gnavus*.

### The clusters define risk groups for severe disease development within the first year

**Figure 5:**
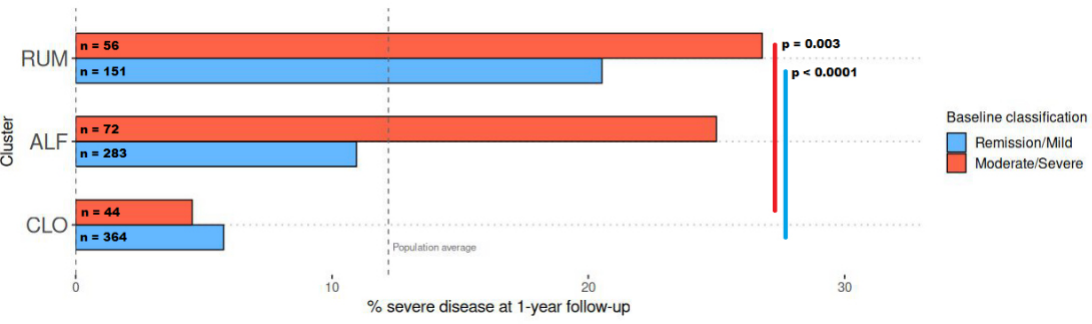
Barplots showing the rates of severe disease course (see Methods for definition) across two categories of baseline disease severity, by cluster. CLO exhibited a relatively low rate of severe disease course (5%-6%), whereas RUM showed a high rate (21%-27%), irrespective of baseline disease severity. Blue = remission/mild disease at baseline, red = moderate/severe disease at baseline (see METHODS for definitions). Figure created with ggplot2.0 in R. CLO:* Clostridia UCG-014*; ALF: *Agathobacter, Lachnoclostridium*, and *Faecalibacterium*; RUM: *Ruminococcus gnavus*.

RUM was associated with the highest risk of a severe disease course in the first year (22%), compared with 14% in ALF and 6% in CLO (chi-square test, *P* = 0.01 and *P* < 0.00001; Table 2). The association between cluster and disease severity was strong, irrespective of baseline severity [Figure 5]. For example, among participants with symptomatic remission or mild disease at inclusion, those in the RUM cluster experienced a 21% rate of severe disease course (*n* = 31 of 151), whereas the corresponding rate in CLO was significantly lower at 6% (chi-square test, *P* < 0.0001, *n* = 21 of 364). 

F-calprotectin and CRP are markers of inflammation associated with more severe outcomes. We investigated cluster assignment, F-calprotectin, and CRP as predictors of severe disease course [Supplementary Table 4]. The two models utilizing either cluster assignment or F-calprotectin alone showed comparable performances, while combining both significantly improved model performance [analysis of variance (ANOVA) with chi-square test, *P* < 0.0001*; *deviance = 23.5 when adding cluster assignment to F-calprotectin]. A similar pattern was observed when excluding samples exposed to antibiotics [Supplementary Table 4]. Overall, cluster assignment was associated with prognosis independently of baseline inflammation levels (*P* < 0.0001; Supplementary Figure 3). 

### Cluster boundaries and relationship to enterotypes

Using GMM clustering, which allows participants to belong partially to multiple groups through soft clustering, we found that 94% of participants were assigned to their respective clusters with high certainty (> 90%). The remaining 55 low-confidence cases were consistently located between clusters when visualized with PCA plots (Figure 4; larger points with black outlines). These cases also tended to have F-calprotectin levels and demographic characteristics intermediate between those of their closest clusters. An exception was observed for the 13 participants located between ALF and RUM, none of whom had a severe disease course [Supplementary Table 5].

**Figure 6:**
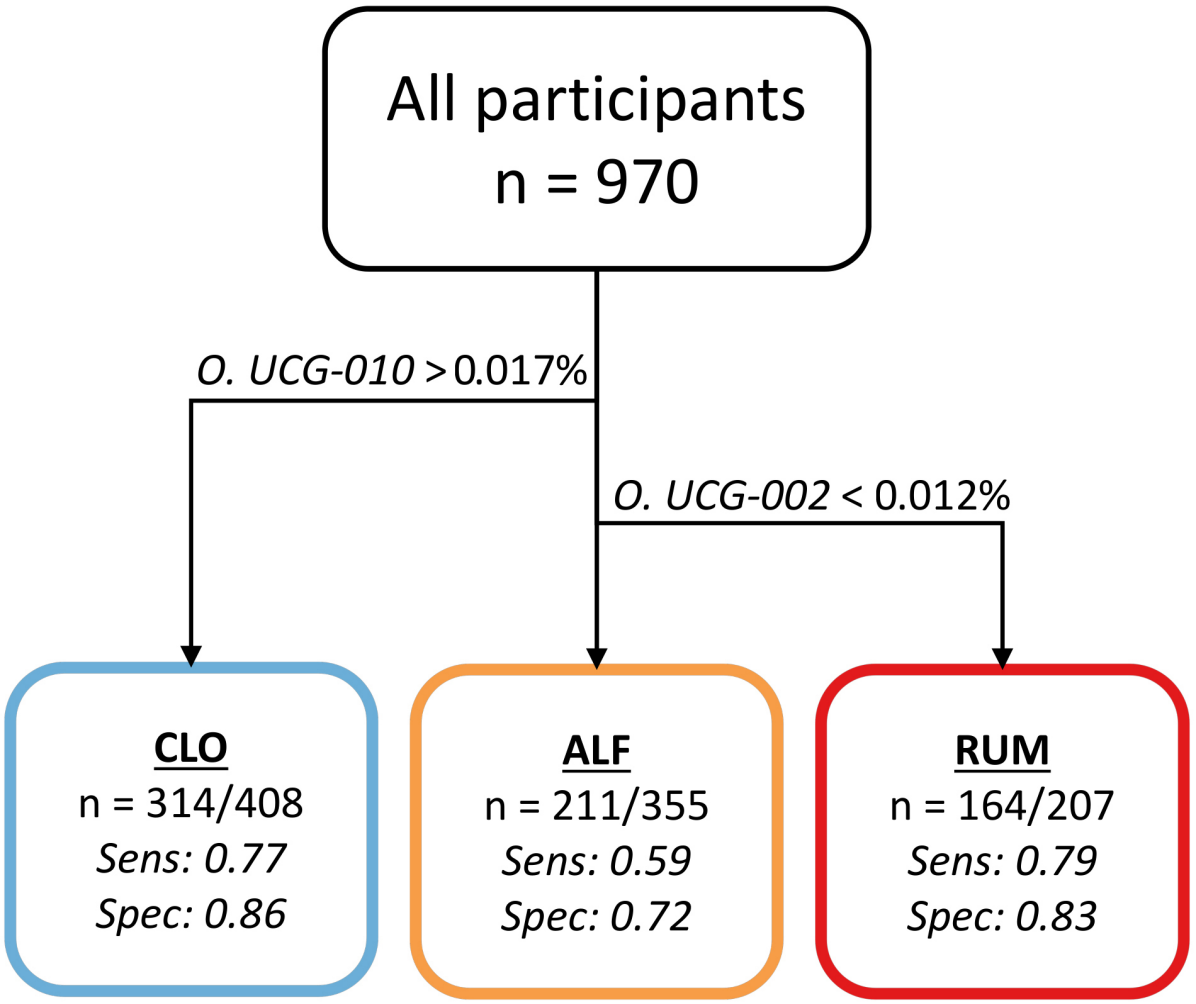
Flowchart illustrating the optimal decision tree with regard to sensitivity (sens.) and specificity (spec.). Samples with O. UCG-010 at a relative abundance > 0.017% are categorized as CLO, with a sens./spec. of 0.77/0.86. Samples with O. UCG-010 below this threshold but O. UCG-002 > 0.012% are categorized as ALF with a sens./spec. of 0.59/0.72. Samples with below-threshold levels of both taxa are categorized as RUM, with a sens./spec. of 0.79/0.83. Figure created in Microsoft PowerPoint.

We used decision trees to describe and explore clusters in a generalizable manner, without overfitting to the current dataset. These trees identify taxa with uniquely high or low abundances in each cluster, thereby identifying taxa with diagnostic properties. Decision tree optimization produced models that separated all clusters with reasonable accuracy using only two taxa: *Oscillospiraceae UCG-002 *and *Oscillospirales UCG-010 *[Figure 6]. Using relative abundance thresholds, samples could be assigned to RUM with a sensitivity of 0.79 and specificity of 0.83. For CLO and ALF, the sensitivity/specificity values were 0.77/0.86 and 0.59/0.72, respectively. 

Finally, we examined the mean abundance of taxa per cluster [Supplementary Table 6]. In CLO and ALF, the most abundant taxon was *Prevotella*. In RUM, *Prevotella *was less abundant and was outnumbered by *Escherichia-Shigella, R. gnavus*, and *Veillonella.* Bacteroides exhibited low abundance across all clusters (0.2%-0.4%). 

## DISCUSSION

In the present study, we used a large population-based IBD cohort to investigate whether unsupervised clustering based on gut microbiome profiles at diagnosis could generate meaningful clinical categories, irrespective of the conventional IBD subtypes. Using GMM, we identified three microbiome-based clusters, to which we could confidently assign 95% of the patients. The clusters differentiated patients with high stability not only at baseline but also in terms of their future risk of severe disease. This approach revealed relatively stable microbial “signatures” that transcend the conventional subtypes of IBD and are not solely explained by the current distribution of inflammation, inflammatory markers, symptom burden, or antibiotic use.

The three clusters defined by our approach were relatively similar in terms of sex, age, distribution of CD and UC, as well as baseline disease activity. However, the three clusters were notably distinct in terms of their microbiome composition and disease course during the first year. For example, Cluster RUM had a particularly high risk of severe disease progression, with *R. gnavus* being its most prevalent taxon. *R. gnavus *is often linked to inflammation and unfavorable disease courses^[31,32]^. Unlike many other taxa in the order Clostridia, *R. gnavus* does not produce butyrate, but may potentially lead to a shift in the microbial ecosystem toward a more inflammatory configuration^[33,34]^. *R. gnavus* thrives on short mucin chains, which may be more abundant or accessible in IBD^[35]^, suggesting that it could serve as a biomarker of an altered gut mucosal environment rather than a direct cause of disease.

Importantly, the associations we uncovered were not simply reflections of pre-existing inflammation. Even when controlling for F-calprotectin, a well-established inflammatory marker, the microbiome-informed clusters offered a meaningful improvement in risk stratification [Supplementary Table 4]. This was particularly evident in participants with remission or mild disease at baseline, where cluster membership separated low-risk (6%) from high-risk (21%) individuals in a manner that conventional inflammation markers or disease activity metrics could not (*P* < 0.00001). Microbial clusters such as those presented in the current study have the potential for improving clinical practice, but follow-up studies with external validation are needed. 

There are few published data available for direct comparison to our study. Previous studies have applied unsupervised clustering methods to identify microbiome-based subgroups in IBD patients, but these clusters were not associated with clinical outcomes such as patients’ risk of progressing to a severe disease state, which our study examines^[36,37]^. Separately, the concept of enterotypes within the general gut microbiome (not specific for IBD) was developed in the seminal gut microbial clustering studies conducted by the international Meta-HIT consortium^[38]^. The enterotype model suggested the existence of distinct microbial compositional “types”, in which individuals (healthy or ill) could be categorized according to their dominant gut bacteria. The concept was controversial due to weak evidence for clear separation^[39]^, but there has recently been renewed interest with improved methods and revised assumptions^[38,40-42]^. Four types are typically described: *Prev *(abundant in *Prevotella*)*, Rum *(abundant in *Ruminococcus *and fiber degraders such as *Faecalibacterium *and *Oscillospiraceae*)*, Bact1 *(abundant in *Bacteroides vulgatus* and *B. uniformis*), and *Bact2 *(abundant in *B. fragilis* and *Escherichia coli*)^[43]^*. *Importantly, the underlying hypothesis of the enterotype model is that these represent relatively stable microbial categories within the global population that may influence disease predisposition. We regard enterotypes as a largely independent theory from our hypothesis. In contrast, we propose that microbiota profiles could be used to create pragmatic but clinically useful clusters with flexible boundaries, specifically within IBD. In other words, we only claim that our described clusters are arbitrary but useful cut-offs along a continuum, rather than distinct “types” as proposed in the enterotype literature.

Enterotype studies are difficult to compare directly with our results. The *Bact2* enterotype is rare in the overall population but may be overrepresented in IBD^[40]^. In our study, *Bacteroides* was not an especially abundant taxon in any of the clusters. Rather, both CLO and ALF seem to fit into the *Prev* enterotype. Vieira-Silva *et al.* described the Rum enterotype as having “low percentages of *Bacteroides* and *Prevotella*”^[38]^. In this respect, both RUM and Rum appear to describe similar community types. However, *Bacteroides* and *Prevotella* still represent the two most abundant taxa in their *Rum* cluster. An important detail is that *R. gnavus* is no longer considered part of the genus *Ruminococcus*^[44]^, but its traditional name is retained in the current article by convention. This means that high abundances of *R. gnavus* do not necessarily indicate increased levels of *Ruminococcus* sensu stricto in the *Rum* enterotype. Unlike previous work, which defines dominant taxa for each cluster or enterotype by averaging relative abundances across samples, our approach avoids bias from isolated cases of high abundance by also considering prevalence (i.e., the proportion of participants in whom the taxon is present). This results in a more balanced and informative selection of dominant taxa for each cluster. Overall, we conclude that our three clusters represent entities that differ from the previously described enterotypes. 

With regard to clustering methods, Vieira-Silva *et al.*^[38]^ and Shi *et al.*^[45]^ used DMM clustering, a probabilistic clustering method similar to GMM. DMM assumes that data points are sampled from multinomial distributions whose probabilities are drawn from a Dirichlet distribution^[45]^. In contrast, GMM assumes that the data points are generated from a mixture of Gaussian distributions and uses the Expectation–Maximization (EM) algorithm to estimate the parameters of each Gaussian component. DMM effectively clusters compositional microbiome data without the need for CLR-transformation, as used in the current paper. However, compared with DMM, GMM is computationally efficient, interpretable, and can incorporate both compositional and non-compositional features (e.g., biochemical markers), which provides a unique advantage for future integrated clustering analyses. These advantages of GMM, together with its highest stability score among all benchmarked methods at k = 3 [Supplementary Tables 2 and 7-11], led us to focus on GMM in the current work. Another potential advantage of GMM, not touched upon in this work, is the possibility of integrating domain knowledge by assigning different weights to taxa expected to play important roles. With the growing body of literature on the relationship between the gut microbiome and IBD, this capability may become increasingly valuable.

Importantly, the results of the present study should not be interpreted as evidence that gut microbiome compositions represent discrete categories. Although cluster stability was high, the clusters identified in this study had low Silhouette scores, an important metric in unsupervised clustering that describes how compact clusters are internally and both the internal compactness of clusters and the degree of separation between clusters^[46]^. Unfortunately, this metric has been omitted in most previous studies on microbiome clusters^[38,40,41,47]^. The first paper proposing the existence of enterotypes reported Silhouette scores around 0.2^[48]^, which is generally not considered high^[46]^, although it is still considerably higher than the values observed in the present study. However, the Silhouette index assumes convex cluster structures, an assumption that might be inappropriate for microbiome sequencing data. We acknowledge that the microbiome may not form distinct clusters^[39]^, but may instead represent points along a continuum. Nevertheless, based on our results, we argue that analyzing putative microbiome clusters can serve as a useful approach for filtering noise and identifying relevant patterns. To account for the absence of clearly defined cluster boundaries, we deliberately applied soft clustering techniques.

The traditional (conventional) classification of IBD into UC and CD remains clinically relevant for risk stratification and the choice of treatment strategies. However, our results suggest that parallel classifications, irrespective of IBD subtype, may also be valuable and could complement the current classification systems. A decision tree analysis helped pinpoint specific bacterial groups that serve as indicators of cluster membership. This was most notable in the RUM cluster, which had a significantly elevated risk of severe disease and could be recognized by a deficiency in two key commensal taxa that are putative short-chain fatty acid producers^[49,50]^: *Oscillospirales UCG-010* and *Oscillospiraceae UCG-002*. This finding indicates potentially powerful clinical utility, since moderate-to-good classification accuracy (sensitivity 0.59-0.79 and specificity 0.72-0.86) can be achieved with only these two taxa [Figure 6]. Additionally, the use of GMMs and their soft clustering capabilities shows the possibility for uncertainty quantification and highlights “in-between” cases that may not fit into traditional diagnostic boxes. This flexibility could be highly advantageous for refining our understanding of less well-defined IBD subgroups, including those described in the literature with unique genetic or environmental signatures^[6]^.

The findings must be interpreted in the context of certain limitations. The results are based on a single population-based inception cohort without validation. Although we leveraged a relatively large sample size and performed bootstrapping procedures to enhance generalizability, the findings should be considered preliminary until validated in independent cohorts. Specifically, independent validation in several cohorts is needed to confirm the clinical relevance of the two taxa identified by the decision tree model. Future studies should also investigate whether incorporating additional biomarkers can improve the decision tree’s classification performance (sensitivity/specificity). Moreover, given the potential risk of misdiagnosis, the clusters themselves must be validated across multiple cohorts and time points to confirm their robustness as biomarkers and to benchmark their performance against existing tools. Additionally, future work should utilize higher-resolution sequencing methods, such as shotgun metagenomic sequencing, to more accurately characterize the taxa involved in the clusters and to investigate the functional aspects of cluster-disease associations.

In conclusion, by moving beyond conventional classifications and incorporating the gut microbiome as a separate dimension defined by unsupervised clustering, we may gain deeper insights into the pathophysiology of IBD, enhance prognostic accuracy, and ultimately guide personalized and targeted therapeutic strategies. 

## DECLARATIONS

### Availability of data and materials

The raw data supporting the conclusions of this article will be made available by the corresponding author upon request.

### Copyright

© The Author(s) 2026.
