# Supplementary material for: Bacterial clusters are associated with the risk of severe disease progression in inflammatory bowel disease irrespective of conventional disease categories
Source: Microbiome Res Rep. 2026 Mar 18;5(1):4. doi: 10.20517/mrr.2025.96 (PMC13092892; doi:10.20517/mrr.2025.96)
Supplement: Supplementary file 1 [file mrr-5-1-4-SupplementaryMaterials.pdf]

Supplementary Materials

Bacterial clusters are associated with the risk of severe disease progression in inflammatory bowel disease irrespective of conventional disease categories

Simen Hyll Hansen[1,2,3,#], Niharika Bhattacharjee[4,#], Chang Hu[5], Maria Gjerstad Maseng[2,6,7], Olle Grannö[8], Corinna Bang[9], Christine Olbjørn[10], Gøri Perminow[11], Jørgen Valeur[2,12], May-Bente Bengtson[13], Svein Oskar Frigstad[14], Svend Andersen[15], Tone Bergene Aabrekk[16], Trond Espen Detlie[17], Andre Franke[9], Vendel A. Kristensen[2,6], Jonas Halfvarson[18], Marte Lie Høivik[2,6], Ravishankar K. Iyer[5,†], Johannes Hov[1,2,3,19,†]

[1]Norwegian PSC Research Center, Department of Transplantation Medicine, Division of Surgery, Inflammatory Diseases and Transplantation, Oslo University Hospital, Oslo 0372, Norway.

[2]Institute of Clinical Medicine, Faculty of Medicine, University of Oslo, Oslo 0372, Norway.

[3]Research Institute of Internal Medicine, Division of Surgery, Inflammatory Diseases and Transplantation, Oslo University Hospital, Oslo 0372, Norway.

[4]Siebel School of Computing and Data Science, University of Illinois Urbana-Champaign, Urbana-Champaign, IL 61820, USA.

[5]Department of Electrical and Computer Engineering, University of Illinois Urbana-Champaign, Urbana-Champaign, IL 61820, USA.

[6]Department of Gastroenterology, Oslo University Hospital, Oslo 0450, Norway.

[7]Bio-Me, Oslo 0349, Norway.

[8]Department of Laboratory Medicine, Clinical Microbiology, Faculty of Medicine and Health, Örebro University, Örebro 70182, Sweden.

[9]Institute of Clinical Molecular Biology, Christian-Albrechts-University of Kiel, Kiel 24118, Germany.

[10]Department of Paediatric and Adolescent Medicine, Akershus University Hospital, Lørenskog 1478, Norway.

[11]Department of Pediatrics, Oslo University Hospital 0372, Oslo, Norway.

[12]Unger-Vetlesen Institute, Lovisenberg Diaconal Hospital 0456, Oslo, Norway.

[13]Department of Gastroenterology, Vestfold Hospital Trust, Tønsberg 3103, Norway.

[14]Department of Medicine, Bærum Hospital, Vestre Viken Hospital Trust, Gjettum 1346, Norway.
[15]Department of Paediatrics, Vestfold Hospital Trust, Tønsberg 3103, Norway.
[16]Medical Department, Vestfold Hospital Trust, Tønsberg 3103, Norway.
[17]Department of Gastroenterology, Akershus University Hospital, Lørenskog 1478, Norway.
[18]Department of Gastroenterology, Faculty of Medicine and Health, Örebro University, Örebro 70182, Sweden.
[19]Section of Gastroenterology, Department of Transplantation Medicine, Division of Surgery, Inflammatory Diseases and Transplantation, Oslo University Hospital, Oslo 0372, Norway.
[#]These authors contributed equally to this work and shared first authorship.
[†]These authors contributed equally to this work and shared senior authorship.

Correspondence to: Dr. Simen Hyll Hansen, Norwegian PSC Research Center, Department of Transplantation Medicine, Division of Surgery, Inflammatory Diseases and Transplantation, Oslo University Hospital, Oslo 0372, Norway. E-mail: simenhyllhansen@gmail.com

ORCID: Simen Hyll Hansen (0000-0003-0156-9875), Niharika Bhattacharjee (0009-0005-6503-7677), Maria Gjerstad Maseng (0000-0003-3053-0267), Olle Grannö (0000-0002-4329-1659), Corinna Bang (0000-0001-6814-6151), Christine Olbjørn (0000-0001-5680-4313), Gøri Perminow (0000-0002-4158-372X), Jørgen Valeur (0000-0001-7193-7069), May-Bente Bengtson (0000-0002-5615-7141), Svein Oskar Frigstad (0000-0001-7841-608X), Svend Andersen (0000-0002-0283-2789), Tone Bergene Aabrekk (0009-0007-4933-5580), Trond Espen Detlie (0000-0002-1576-5298), Andre Franke (0000-0003-1530-5811), Vendel Ailin Kristensen (0000-0002-0876-8634), Jonas Halfvarson (0000-0003-0122-7234), Marte Lie Høivik (0000-0002-0104-465X), Johannes Hov (0000-0002-5900-8096)

Methods

Partition Around Medoids (PAM)

This clustering method partitioned the data points into a fixed number of clusters through a two-phase process: BUILD and SWAP. During the BUILD phase, k medoids were chosen from the dataset. During the SWAP phase, the PAM method aimed to improve the medoid set

by accepting a swap between the cluster medoid and a candidate non-medoid if the cluster had a lower total sum of dissimilarities with the candidate as the new medoid. This process of assigning points to their closest medoid after each accepted swap, followed by further swaps, continued until convergence. At the end of clustering, the final medoids were the most centrally located datapoints in their respective clusters.

Agglomerative Clustering (Ward's Method)

Referred to as a bottom-up clustering method, this approach began by placing each data point in its own individual cluster. Based on the specified linkage criterion, the pair of clusters were merged to form a new cluster. Specifically, in our case, the Ward linkage criterion was applied, which stated that clusters should be merged based on the pair whose merge resulted in the smallest increase in the total within-cluster variance. This process was repeated until one cluster remained.

Spectral Clustering

Known as a graph-based clustering method, this approach grouped the data points into k clusters by first constructing a similarity graph (e.g., a KNN graph). The k smallest eigenvalues from the similarity graph's Laplacian matrix were used to create a feature matrix of corresponding eigenvectors. K-means clustering was then applied to this feature matrix to identify distinct groups.

Gaussian Mixture Model (GMM) clustering

This distribution-based clustering method modeled the data as a mixture of several Gaussian distributions. Each cluster was represented by a mixture component k defined by parameters, [Mixing coefficient π_k, Mean μ_k, Covariance Σ_k], whose values represent the proportion of data that belonged to cluster k, the mean vector for cluster k, and the covariance matrix for cluster k. During the E-step of the Expectation-Maximization (EM) algorithm, the probability of a data point belonging to each cluster was computed using the parameters of the given cluster. These probabilities across all the data points were used to update each cluster's parameters during the M step of the EM algorithm. This clustering method allowed for soft assignments of data points to clusters based on their likelihood of belonging to each cluster.

Supplementary Tables and Figures

Supplementary Table 1. Comparison of participants eligible and not eligible for inclusion in the study

	Included (N=970)	*Not included (N=151)*
Age		
Mean (SD)	36.9 (16.7)	40.6 (17.9)
Median [Min, Max]	35.0 [3.0, 82.0]	41.0 [4.0, 79.0]
Sex (female)	504 (52%)	76 (50%)
BMI		
Mean (SD)	24.7 (5.49)	25.1 (5.62)
Median [Min, Max]	24.0 [12.0, 68.0]	24.0 [12.0, 47.0]
Fecal calprotectin		
Mean (SD)	575 (655)	650 (705)
Median [Min, Max]	241 [29.0, 1800]	292 [29.0, 1800]
C-reactive protein		
Mean (SD)	10.7 (22.1)	15.4 (46.9)
Median [Min, Max]	3.00 [0, 185]	3.00 [0.5, 280]
Diagnosis		
Ulcerative colitis	625 (64%)	86 (57%)
Crohn's disease	345 (36%)	65 (43%)
Severe disease course*	118 (12%)	31 (20%)
Missing	0 (0%)	58 (38.4%)
Hospitalization		
Mean (SD)	0.0866 (0.281)	0.116 (0.323)
Median [Min, Max]	0 [0, 1.00]	0 [0, 1.00]
Missing	0 (0%)	82 (54.3%)
Symptom Score**		
Mean (SD)	0.637 (0.795)	0.615 (0.813)
Median [Min, Max]	0 [0, 3.00]	0 [0, 3.00]
Missing	0 (0%)	55 (36.4%)
Sampling Delay***		
Mean (SD)	50.8 (91.0)	43.5 (77.3)
Median [Min, Max]	22.0 [-334, 959]	19.0 [1.0, 544]
Missing	2 (0.2%)	22 (14.6%)

Demographic and clinical characteristics of IBSEN III participants with IBD included and not included in this study. *Severe disease course during the first year after inclusion (see Methods for definition); **Integrated score, 0 to 3 (see Method for definition); ***Number of days from inclusion to fecal samples delivered. SD – Standard Deviation; BMI – Body Mass Index

Supplementary Table 2. Summary of clustering methods that were benchmarked

**Clustering Method**	**Important Attributes**	**Silhouette Index**	**Cluster Stability**	**Type of Clustering**
GMM Clustering	Covariance Type: Spherical; Input type: Compositional	0.06	**0.74**	Distribution-based
K-Medoids	Distance: Aitchison; Method: PAM; Input Type: Compositional	0.05	0.48	Partitioning-based
Agglomerative Clustering	Distance: Aitchison; Linkage: Ward; Input Type: Compositional	0.07	0.38	Hierarchical
Spectral Clustering	Affinity Metric: Nearest Neighbors;	**0.08**	0.60	Graph-based

	Input Type: Compositional			
DMM Clustering	Input type: Raw Count	0.04	0.72	Distribution-based

Benchmarking table for tested clustering methods, presenting statistics for k=3. Due to low silhouette index scores across all methods, cluster stability was used to select the preferred clustering method. Abbreviations: GMM – Gaussian mixture models; PAM – Partition Around Medoids; DMM – Dirichlet Multinomial Mixtures; BIC – Bayesian Information Criterion; AIC – Akaike Information Criterion.

Supplementary Table 3. Statistical summary and pair-wise cluster comparisons of key clinical characteristics

	**Test**	**Test statistic**		**p-value**		**Post hoc**	**Cluster-specific results***		
Variable	**method**	**Antibiotics included**	**Antibiotics excluded**	**Antibiotics included**	**Antibiotics excluded**	**analysis**	**Comparison**	**Antibiotics included**	**Antibiotics excluded**
Age at inclusion	*Kruskal-Wallis*	48.29	51.20	< 0.0001	< 0.0001	*Dunn's Test (p-values)*	CLO vs ALF	< 0.0001	< 0.0001
							ALF vs RUM	0.65	0.93
							CLO vs RUM	< 0.0001	< 0.0001
Weight (kg)	*Kruskal-Wallis*	0.66	0.58	0,72	0,75	-		-	-
Bristol Stool Scale	*Kruskal-Wallis*	16.00	10.78	0,0003	0,0046	*Dunn's Test (p-values)*	CLO vs ALF	0.17	0.071
							ALF vs RUM	0.08	0.80
							CLO vs RUM	0.0002	0.0076
*Antibiotics***	*Chi-Squared*	62.04	-	< 0.0001	-	*Proportion analysis (residuals of standard deviation)*	CLO	-4.97	-
							ALF	-1.44	-
							RUM	7.68	-
*Baseline severity****	*Kruskal-Wallis*	25.23	24.85	< 0.0001	< 0.0001	*Dunn's Test (p-values)*	CLO vs ALF	0.05	0.03
							ALF vs RUM	0.011	0.016
							CLO vs RUM	< 0.0001	< 0.0001

*Severe course*** **	*Chi-Squared*	36.75	35.36	< 0.0001	< 0.0001	*Proportion analysis (residuals of standard deviation)*	CLO	-5.3	-5.3
							ALF	1.2	1.64
							RUM	5.0	4.7

*Statistical details on key demographic and clinical variables, before and after excluding samples with antibiotic exposure. *Significant Pairwise Comparisons (Adjusted p-values) OR cluster-specific proportion analysis (standard deviations from expected proportions given the null hypothesis); **Antibiotics within 3 months prior to study inclusion; ***Combined categorization of Simple Clinical Colitis Index and Harvey-Bradshaw Index, see Methods; ****Severe disease course during the first year after inclusion, see Methods.* Abbreviations: Kg – kilogram; CLO/ALF/RUM – Names of clusters defined in the current work.

Supplementary Table 4. Statistical summary of models predicting severe disease course

	Antibiotics included				*Antibiotics excluded*			
Model	**AIC**	**BIC**	**R^2**	**AUC**	**AIC**	**BIC**	**R^2**	**AUC**
Model 1	687	702	0.051	0.659	606	620	0.056	0.665
Model 2	687	697	0.049	0.658	606	615	0.052	0.658
Model 3	711	721	0.015	0.652	633	643	0.009	0.651
Model 4	**667**	**687**	0.082	0.710	**587**	**606**	**0.088**	0.722
Model 5	685	704	0.058	0.690	606	625	0.058	0.700
Model 6	686	700	0.053	0.679	607	622	0.053	0.667
Model 7	668	693	**0.083**	**0.722**	589	613	**0.088**	**0.724**

Statistical summary of differing models evaluated using binomial generalized linear models (GLMs) to classify severe disease course. Model 1 – Clusters; Model 2 – Fecal calprotectin (F-Cal); Model 3 – C-reactive protein (CRP); Model 4 – Cluster + F-Cal; Model 5 – Cluster + CRP; Model 6 – F-Cal + CRP; Model 7 – Cluster + F-Cal + CRP. The best-performing model per column is marked with bold text. The overall best model was Model 4, based on minimizing AIC and BIC while maintaining R^2 and AUC at a close second to Model 7. In Model 4, both Cluster and F-Cal were significantly associated with severe disease course ($p < 0.0001$ for both). Abbreviations: BIC – Bayesian Information Criterion; AIC – Akaike Information Criterion; AUC – Area Under the receiver operating Curve.

Supplementary Table 5. Summary of participants belonging to more than one cluster

**Primary cluster**	**Secondary cluster**	**n**	**Age**	**Sex (male %)**	**F-cal.***	**Severe***
ALF (all)		***-***	***33***	***56%***	***675***	***13.8%***
ALF	CLO	20	40	55%	403	10%

ALF	RUM	7	41	14%	385	0%
CLO (all)		**-**	***41***	***45%***	***393***	***5.6%***
CLO	ALF	21	45	52%	668	5%
CLO	RUM	1	29	100%	190	100%
RUM (all)		**-**	***35***	***42%***	***770***	***22.2%***
RUM	ALF	6	35	67%	888	0%

Simple summary of the participants (n=55) who could not be assigned to any cluster with confidence >90%. With regard to inflammation levels and prevalence of a severe disease course, these edge cases are intermediate or closer to the characteristics of their primary cluster. An exception is the lack of severe disease course in participants intermediate between ALF and RUM (n=13), where there were no cases of severe disease course. *Fecal calprotectin (mean); **Severe disease course during first year after inclusion. Abbreviations: n – number of participants; CLO/ALF/RUM – Names of clusters defined in the current work.

Supplementary Table 6. Summary of dominant taxa per cluster

**CLO**	**ALF**	**RUM**
Prevotella (9%)	*Prevotella (10%)*	*Escherichia-Shigella (8%)*
Muribaculaceae (5%)	*Phascolarctobacterium (4%)*	*Ruminococcus gnavus (8%)*
Ruminococcaceae CAG-352 (4%)	*Escherichia-Shigella (3%)*	*Veillonella (5%)*
Clostridia UCG-014 (4%)	*Muribaculaceae (2%)*	*Prevotella (5%)*

Top 4 most abundant taxa per cluster, as measured by the mean of relative abundances. Abbreviations: CLO/ALF/RUM – Names of clusters defined in the current work.

Supplementary Table 7. Benchmarking of agglomerative clustering

**k**	2	3	4	5	6	7	8
**Silhouette**	0.09	0.08	0.04	0.04	0.04	0.03	0.03
**Stability**	0.45	0.39	0.34	0.34	0.33	0.33	0.33

An extended benchmarking table for Agglomerative Clustering, presenting statistics for k=2 to k = 8.

Supplementary Table 8. Benchmarking of K-Medoids

**k**	2	3	4	5	6	7	8
**Silhouette**	0.07	0.05	0.03	0.03	0.03	0.03	0.02
**Stability**	0.43	0.48	0.40	0.35	0.39	0.37	0.35

An extended benchmarking table for K-Medoids, presenting statistics for k=2 to k = 8.

Supplementary Table 9. Benchmarking of spectral clustering

**k**	2	3	4	5	6	7	8
**Silhouette**	0.14	0.09	0.05	0.05	0.05	0.04	0.04
**Stability**	0.73	0.60	0.54	0.48	0.44	0.45	0.39

An extended benchmarking table for Spectral Clustering, presenting statistics for k=2 to k = 8.

Supplementary Table 10. Benchmarking of Gaussian mixture model clustering

**k**	2	3	4	5	6	7	8
**Silhouette**	0.10	0.06	0.05	0.05	0.04	0.03	0.03
**Stability**	0.83	0.74	0.52	0.48	0.47	0.43	0.45

An extended benchmarking table for the Gaussian Mixture Model, presenting statistics for k=2 to k = 8.

Supplementary Table 11. Benchmarking of Dirichlet-multinomial mixture clustering

**k**	2	3	4	5	6	7	8
**Silhouette**	0.04	0.04	0.03	0.02	0.01	0.01	0.01
**Stability**	0.80	0.70	0.83	0.75	0.64	0.59	0.52

An extended benchmarking table for Dirichlet-Multinomial Mixture (DMM) clustering, presenting statistics for k=2 to k = 8.

Supplementary Figures

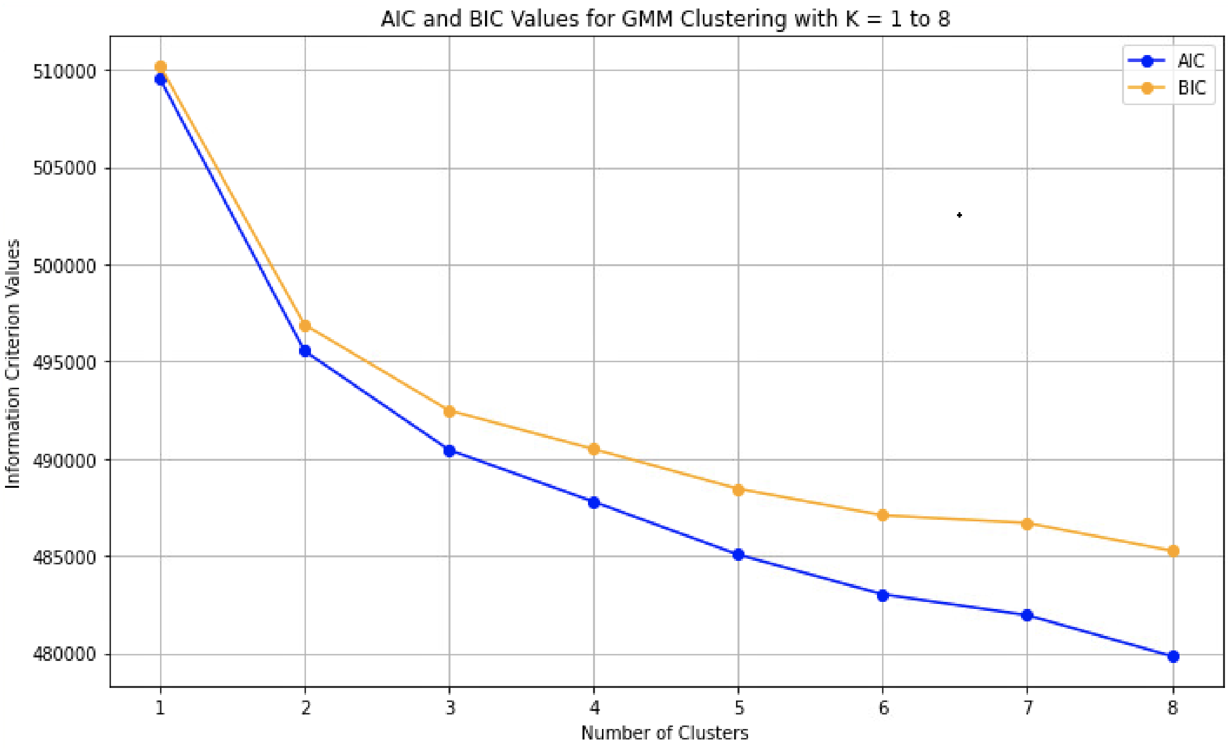

Supplementary Figure 1. AIC and BIC curves for the number of clusters from k=1 to 8 for GMM. Abbreviations: BIC – Bayesian Information Criterion; AIC – Akaike Information Criterion.

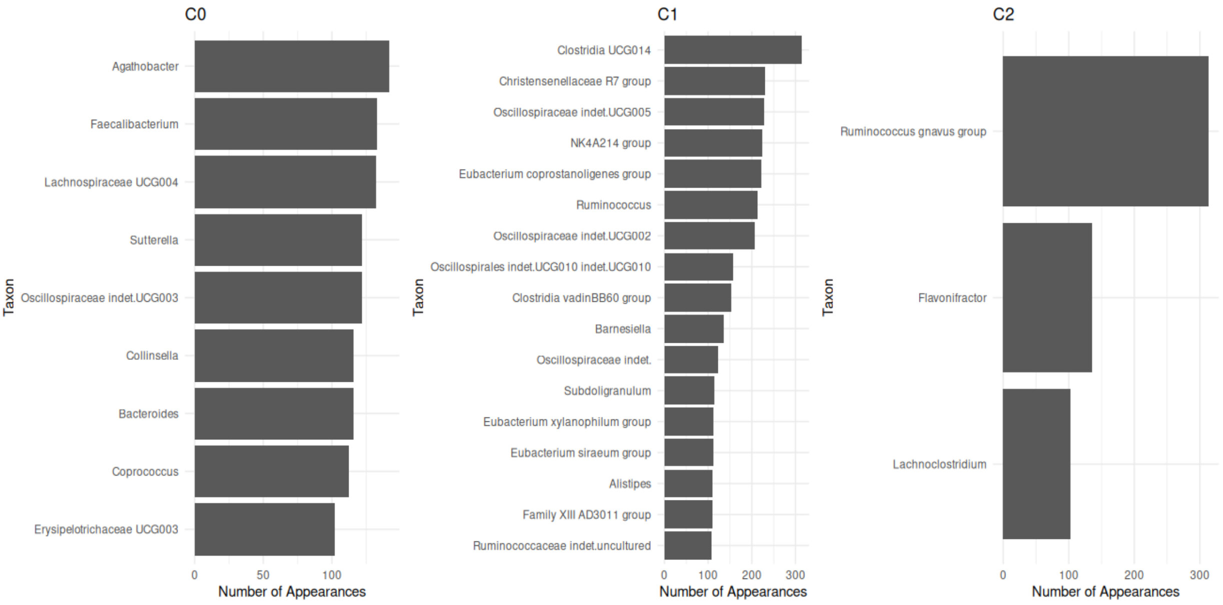

Supplementary Figure 2. A repeat analysis with the same iterative procedure as in Figure 1, except that samples with antibiotic exposure are excluded from the procedure. C0 refers to ALF, C1 to CLO, C2 to RUM.

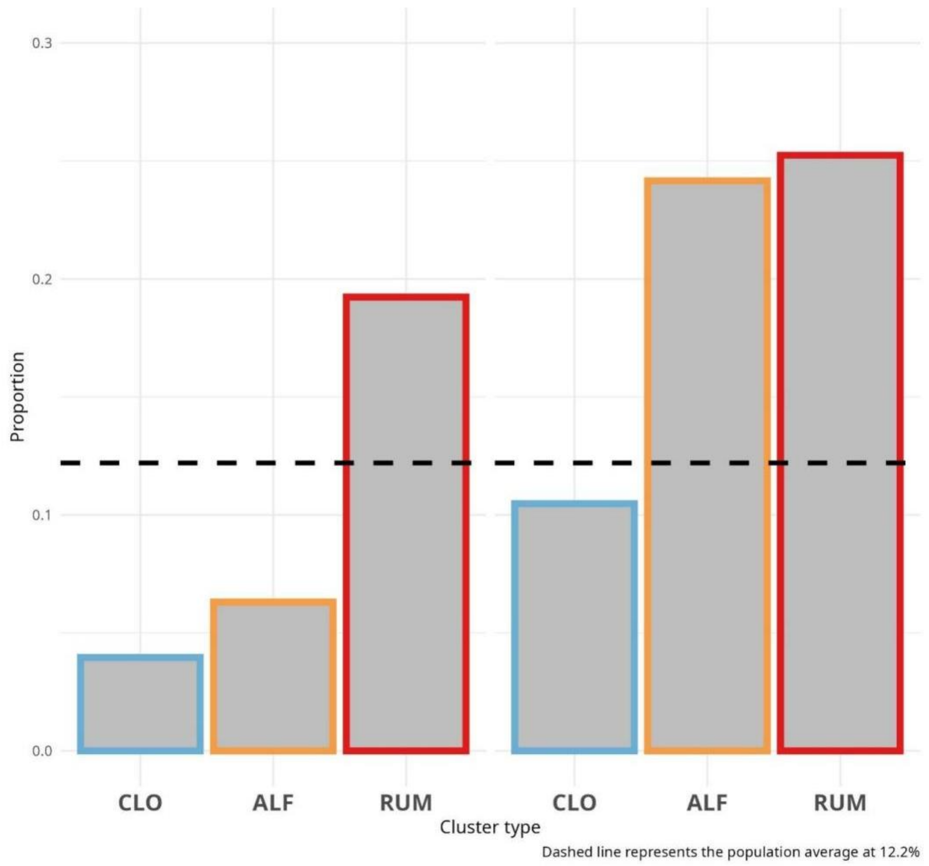

Supplementary Figure 3. Left: below-median fecal calprotectin (< 433); right: above-median fecal calprotectin (> 433). As shown in Supplementary Table 4, cluster assignment is significantly associated with prognosis when controlling for fecal calprotectin (ANOVA, $P < 0.0001$).
